# Sex-specific intergenerational epigenetic responses to real-world chemical mixture exposure in an outbred sheep model

**DOI:** 10.1093/pnasnexus/pgag338

**Published:** 2026-10-10

**Authors:** Oliver G Hargreaves, Wing Yee Kwong, Andrew Warry, Desmond A R Tutt, Vasantha Padmanabhan, Neil P Evans, Richard G Lea, Michelle Bellingham, Kevin D Sinclair

**Affiliations:** Schools of Biosciences and Veterinary Medicine and Science, University of Nottingham, Sutton Bonington LE12 5RD, United Kingdom; Schools of Biosciences and Veterinary Medicine and Science, University of Nottingham, Sutton Bonington LE12 5RD, United Kingdom; Schools of Biosciences and Veterinary Medicine and Science, University of Nottingham, Sutton Bonington LE12 5RD, United Kingdom; Schools of Biosciences and Veterinary Medicine and Science, University of Nottingham, Sutton Bonington LE12 5RD, United Kingdom; Department of Pediatrics, University of Michigan, Ann Arbor, MI 48109-5718, USA; School of Biodiversity, One Health and Veterinary Medicine, University of Glasgow, Glasgow G61 1QH, United Kingdom; Schools of Biosciences and Veterinary Medicine and Science, University of Nottingham, Sutton Bonington LE12 5RD, United Kingdom; School of Biodiversity, One Health and Veterinary Medicine, University of Glasgow, Glasgow G61 1QH, United Kingdom; Schools of Biosciences and Veterinary Medicine and Science, University of Nottingham, Sutton Bonington LE12 5RD, United Kingdom

**Keywords:** environmental chemicals, DNA methylation, epigenetic inheritance, genetics, sexual dimorphism

## Abstract

Establishing whether real-world environmental chemical (EC) exposure can induce heritable epigenetic modifications in large, outbred mammals is a key to determining long-term developmental impacts of the human exposome. Using an established biosolid-treated pasture sheep model, we investigated whether gestational exposure to low-level mixtures of EC induced heritable changes in DNA methylation across three generations of sheep. Reduced-representation bisulfite sequencing of liver, blood, and sperm, combined with a structured, lineage-controlled breeding design, revealed widespread but lineage- and sex-specific differentially methylated loci (DML) in F1 offspring, with detectable alterations evident in F2 and F3 descendants. Although most DML were unique to individual sire lineages or to a single generation, subsets of loci showed repeated involvement across generations and were associated with altered gene expression in F3 descendants. Sperm from F1 males exhibited reduced methylation at numerous loci and, together with seminal plasma, revealed differential expression of several microRNAs. These effects, however, showed limited persistence in F2 males, indicative of intergenerational rather than fully transgenerational persistence. Collectively, these findings demonstrate that complex, low-level chemical exposures can elicit recurrent, sexually dimorphic epigenetic responses in outbred species but underscore the challenge of disentangling exposure-induced inheritance from genetically regulated methylation variation.

Significance Statement Environmental chemical (EC) exposures are ubiquitous, yet their capacity to induce heritable epigenetic changes in large, genetically diverse mammals is poorly understood. Using a real-world exposome-based sheep model, we demonstrate that low-level gestational EC exposure leads to sexually dimorphic and lineage-dependent alterations in DNA methylation that can extend to unexposed descendants. Although genetic ancestry exerts a dominant influence over these responses, repeated alterations at specific loci suggest that environmentally induced epimutations can reoccur across generations in certain genomic contexts.

## Introduction

Exposure to complex mixtures of environmental chemicals (ECs) prior to and throughout pregnancy is of concern in developmental toxicology, particularly due to the potential for long-lasting effects on offspring reproductive and metabolic health (1, 2). In this regard, epigenetic modifications to DNA methylation provide a compelling mechanism by which such exposures can exert their influence, with the potential to extend across generations (3, 4). The extent to which low-level EC exposure during gestation can alter the epigenome in large, precocial outbred mammals like humans, and whether these modifications can be inherited, remains unknown. A powerful model for studying these issues involves grazing pregnant sheep on pastures treated with biosolids (BSs), a processed by-product of human sewage treatment that is used globally as an agricultural fertilizer (5). BSs contain low concentrations of a mixture of anthropogenic contaminants (including phthalates, per- and polyfluoroalkyl substances, flame retardants, pharmaceuticals, and organochloride pesticides), rendering this model representative of the human exposome both in terms of composition and concentration of EC (6).

Early studies using the BS sheep model revealed a range of adverse effects on the fetal and adult hypothalamic–pituitary–gonadal axis (7–11). More recent BS studies working with the cohort of sheep reported in the current article describe a testicular dysgenesis-like phenotype in F1 juvenile and adult male offspring similar to that reported in humans (12–14), effects on puberty onset in both sexes (15), and coincident evidence of reduced ovarian reserve in juvenile females (16, 17). Metabolic studies have also demonstrated dysregulation in both maternal (F0) and adult F1 offspring, with evidence of inter-individual variability in responses to EC exposure (18–21).

To date, there has been no investigation of the epigenetic mechanisms that could underlie these phenotypic effects within the BS model or in large-animal models of EC exposure more generally (1, 22). As such, it is unknown whether gestational exposure to EC from BS induces DNA methylation changes in offspring and whether such effects can be transmitted to later nonexposed generations. Elucidation of such heritable epigenetic changes in this model would be informative, given the physiological similarities between sheep and humans, and the outbred, genetically diverse nature of sheep as a model species. Evidence of heritable changes in DNA methylation following exposure to EC is limited currently to studies with rodents and to single or small mixtures of chemicals (e.g. vinclozolin, bisphenol A, and phthalates) (3, 23). The investigation of inheritance of EC-induced epigenetic marks in an outbred mammalian species, however, presents challenges. First, genetic variation within a population can confound the attribution of inherited differences in DNA methylation to exposure rather than underlying genotype (24, 25). Furthermore, DNA methylation undergoes two rounds of global reprogramming during early embryogenesis and gametogenesis (26), rendering stable transmission of acquired marks inherently difficult (27). Finally, sexual dimorphism in epigenetic responses following EC exposure (28, 29) must also be factored into any analysis.

With the foregoing discussion in mind, the present study sought to determine whether gestational exposure to a real-life mixture of EC using the BS sheep model would induce heritable changes in DNA methylation that would span three generations of offspring and include the third (F3) nonexposed generation. Reduced-representation bisulfite sequencing (RRBS) was performed on descendant DNA extracted from liver (F1, F2, and F3), sperm (F1 and F2), and blood (F2). In addition, RNA sequencing, with a focus on microRNA, was performed on sperm and seminal plasma from F1 and F2 male offspring, to provide an insight into putative “reconstructive” modes of male germline epigenetic inheritance (27, 30). Consideration was given throughout to the breeding structure of each of the three descendent populations to (i) standardize genetic variation between treatment groups within generation, (ii) avoid family matings, and (iii) facilitate the tracking of differentially methylated loci (DML) across generations.

## Results

### Fertility and pregnancy outcomes

The breeding schedule devised for this study (Fig. S1) generated genetically matched (across the two experimental treatments) family groups for generations F1, F2, and F3. There was no effect of BS exposure of F0 ewes during the 6 weeks leading up to artificial insemination (AI) and gestation on pregnancy establishment, litter size, term delivery, and sex distribution of offspring (Tables S1 and S4). The same was true for F1 and F2 inseminations, leading to the birth of F2 and F3 offspring (Tables S2, S3, S5, and S6).

### F1 epigenetic effects—following EC exposure of pregnant F0 ewes

#### Liver DNA methylation

Samples were collected 8 weeks after birth from 16 F1 male and female offspring, representing the four half-sib family groups for each of the two treatments (Table S4). RRBS reads covered 13,605,795 methylated cytosines, consisting of 83.1% CpG, 5.8% CHG, and 11.1% CHH, with average methylations of 50.9% CpG, 1.9% CHH, and 2.3% CHG (a distribution repeated across all subsequent generations). Note, CpG is a cytosine followed by guanine (C–G), CHG is a cytosine followed by any nonguanine base then guanine (C–H–G), and CHH is a cytosine followed by two nonguanine bases (C–H–H), with H representing adenine (A), cytosine (C), or thymine (T). Hierarchical clustering of CpGs (only) revealed a strong paternal (sire) influence within which were nested the effects of offspring sex and exposure treatment, respectively (Fig. 1A). Subsequent analyses of DML (i.e. only CpGs ≥15% difference [DML]) were performed in two sets, one partitioned by sire (differences averaged across sex) and one partitioned by sex (differences averaged across sire). When partitioned by sire, 25,374 unique DML were observed, 93.4% of which were specific to a single sire (Fig. 1B and F). When partitioned by sex, 3,725 DML were observed with 98.9% specific to a single sex (Fig. 1C and F). Mean methylation differences between treatments, averaged across sire, were similar quantitatively for male (6.9% net increase in BS exposed; Fig. 1D) and female (7.4% net increase in BS; Fig. 1E) offspring. Further analyses identified 502 unique genes, containing genic (i.e. from 3 kb upstream of the transcription start site to 3 kb downstream of the 3-prime untranslated region (3′UTR)) DML, 42.1% of which were specific to males and 54.5% specific to females (File S1A and B).

**Figure 1:**
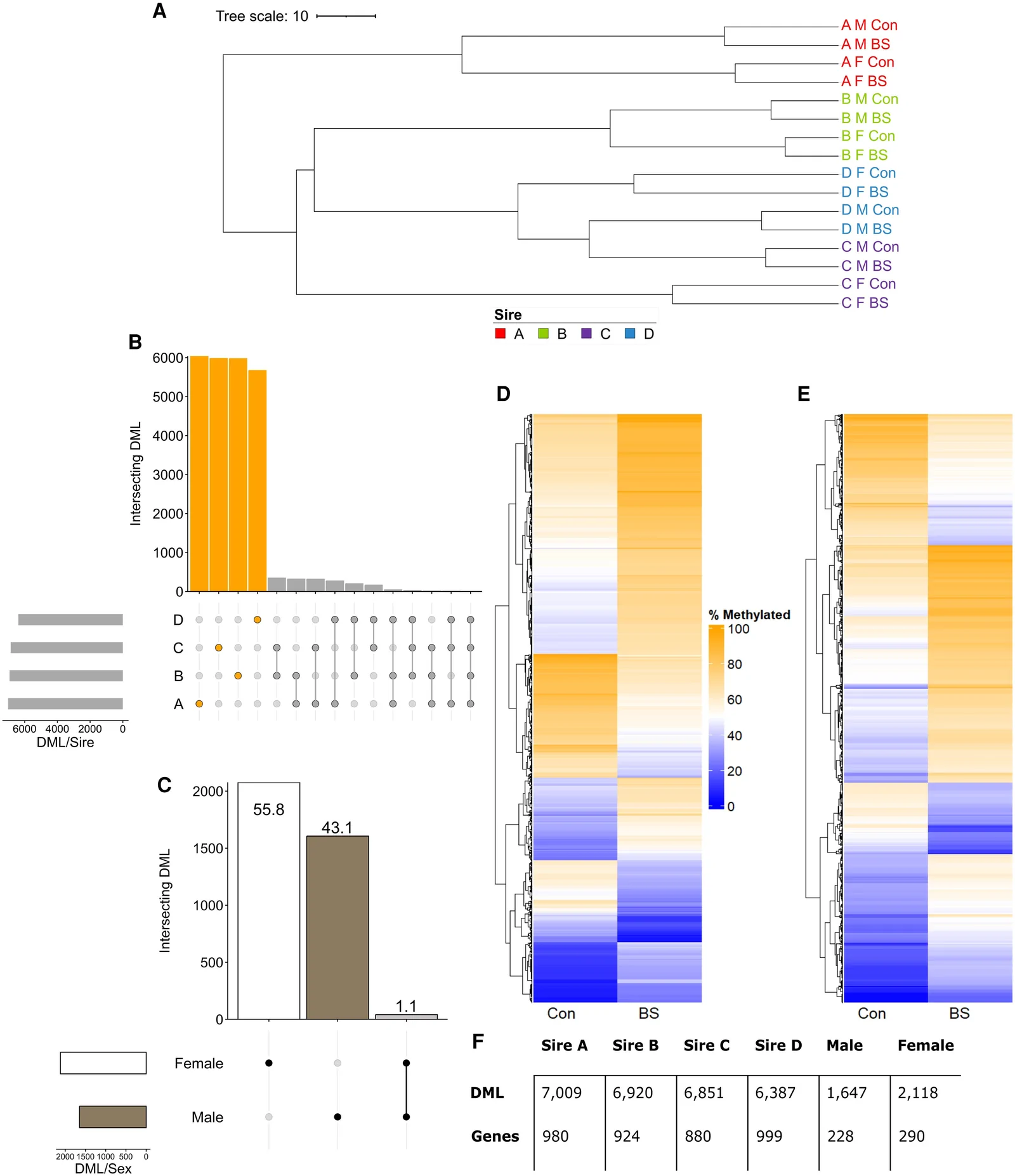
Methylation characteristics of F1 liver. A) Hierarchical clustering of DNA methylation in F1 liver samples. Distances calculated from top three principal components. Label colors indicate sire groupings. B) Upset plot of DML (15% threshold) intersects across the four F1 sire groups. C) Upset plot of DML intersects across male and female F1 livers (overlapping, values on bars denote percentage of total DML). D) Heat map highlighting mean methylation of DML in males in the control (Con) and BS exposed experimental groups. E) Heat map highlighting mean methylation of DML in females in the Con and BS exposed experimental groups. F) DML and DML-containing gene counts in F1 livers across the four sire groups and in males and females.

#### Sperm motility and epigenetics

Semen was collected from 16 sexually mature F1 rams (eight control [Con] and eight BS), representing the four sire family groupings (Table S4). Computer-assisted sperm assessment (CASA) found no effect of in utero BS exposure on either F1 (4.0 ± 0.53 vs. 4.3 ± 0.36 billion/mL, Con vs. BS) or F2 (2.3 ± 0.45 vs. 1.5 ± 0.40 billion/mL, Con vs. BS) sperm concentration. However, BS exposure increased (*P* = 0.044) the percentage of progressively motile F1 sperm (Fig. S2A) as well as F1 sperm straight-line velocity (86.7 ± 6.87 vs. 105.9 ± 4.88 µm/s, Con vs. BS; *P* = 0.042).

For F1 sperm, RRBS reads covered 16,777,158 methylated cytosines. Hierarchical clustering of methylated CpGs revealed a strong sire influence within which was nested treatment (Fig. S3A). Subsequent analyses of DML partitioned by sire revealed 47,921 unique DML, 80.5% of which were specific to a single sire. When averaged across sire lineages, 1,350 unique DML were observed, with a net decrease of 7.2% following BS exposure (Fig. S2B). Further analyses identified 150 genes containing genic DML, 12 of which have recognized roles in testis/sperm function (File S1C).

Micro RNAs (miRNAs) sequenced from seminal plasma and sperm were aligned to 153 mature sequences arising from the miRBase database prior to differential expression (DE) analyses (log_2_ fold change ≥2.0; false discovery rate (FDR) ≤ 0.01). When partitioned by sire, analyses identified six unique miRNAs (miR-376e-5p, miR-376b-5p, miR-362, miR-544-5p, miR-539-5p, and miR-376d). All six were DE in seminal plasma distributed across sire groups. miR-376b-5p was also DE in sperm.

### F2 epigenetic effects—following EC exposure of pregnant F0 ewes

#### Liver DNA methylation

DNA was analyzed from 15 males and 16 females (Table S5). RRBS covered 6,811,089 methylated cytosines. Hierarchical clustering of methylated CpGs revealed a stronger influence of sex, with treatment and grandsire effects nested within, than observed in F1 liver (Fig. S3C). When partitioned by grandsire lineage, 50,700 DML were observed, with 85.6% being specific to a single lineage (Fig. 2A) and with 1,794 DML (1,125 genic) common to those in F1 sperm. When partitioned by sex, 1,256 DML were observed, with 98.2% specific to a single sex (Fig. 2B) and 13 DML (five genic) common to those in F1 sperm. Mean methylation differences between treatments differed between sexes, with a 0.4% net decrease in males (Fig. 2C) and a 4.2% net increase in females (Fig. 2D) following BS exposure. Further analyses identified 174 unique genes containing genic DML, of which 48.3% were specific to males and 49.4% specific to females (File S1E and F).

**Figure 2:**
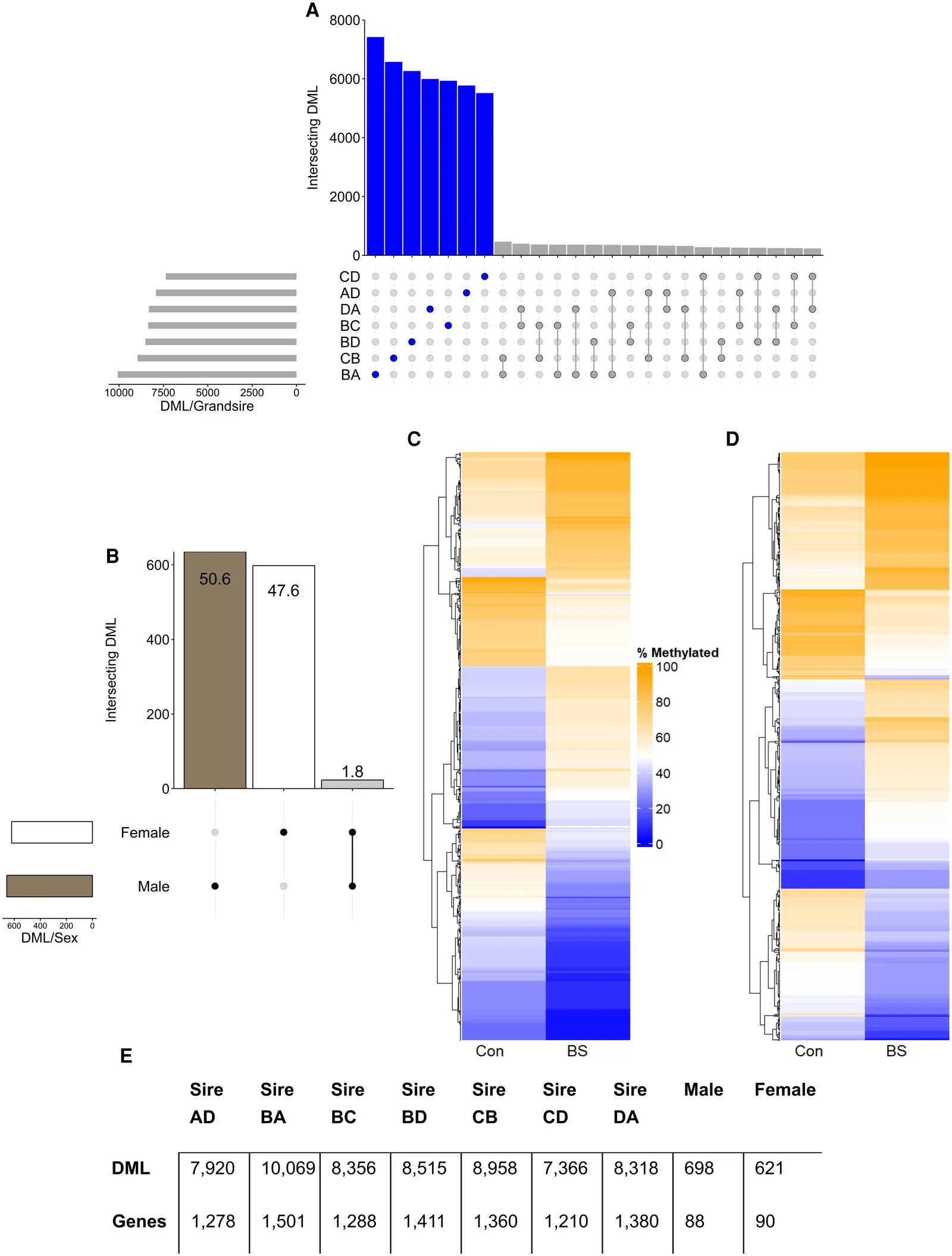
Methylation characteristics of F2 liver. A) Upset plot of DML (15% threshold) intersects across seven F2 grandsire groups. Only the top 25 intersections are displayed. B) Upset plot of DML intersects across male and female F2 liver (values on bars denote percentage of total DML). C) Heat map highlighting mean methylation of DML in males in the control (Con) and BS exposed experimental groups. D) Heat map highlighting mean methylation of DML in females in the Con and BS exposed experimental groups. E) DML and DML-containing gene counts across seven F2 grandsire groups as well as in males and females.

#### Blood DNA methylation

DNA was also analyzed from 22 male and 22 female blood samples, representing almost all possible sire group combinations, balanced across the two treatments (Table S5). RRBS analyses covered 20,596,062 methylated cytosines. Hierarchical clustering of methylated CpGs revealed a similar pattern to F2 liver samples, showing treatment and grandsire lineage effects nested within strong sex effects (Fig. S3D). Differential methylation analyses identified 76,059 DML when partitioned by grandsire, of which 71.7% were specific to a single grandsire (Fig. S4A). When partitioned by sex, 1,650 DML were observed, of which 56.7 and 39.0% were unique to male and female, respectively (Fig. S4B). Mean methylation differences between treatments, when averaged across the grandsire lineage, differed between sexes, with a 10.1% net decrease in males (Fig. S4C) and a 3.0% net increase in females (Fig. S4D) following BS exposure. Further analyses of the 1,650 DML identified 181 unique genes containing genic DML, with 52.5 and 38.7% unique to male and female F2 offspring, respectively (File S1G and H).

#### Sperm motility and epigenetics

Analyses were undertaken in semen from 14 sexually mature F2 rams (seven Con and seven BS), representing seven matched grandsire family groupings (Table S5). In contrast to F1 sperm, there was no effect of BS exposure on percentage of progressive motility of F2 sperm (Fig. S2C) or on F2 sperm straight-line velocity (97.8 ± 14.85 vs. 68.1 ± 13.29 µm/s, Con vs. BS). RRBS reads covered 14,876,916 methylated cytosines. Here, hierarchical clustering of methylated CpGs revealed a strong grand-paternal lineage influence within which was nested treatment (Fig. S3B). When averaged across grandsire lineages, 2,954 unique DML were observed, with a net increase of 2.2% following BS exposure (Fig. S2D). Further analyses identified 197 genes containing genic DML, 15 of which have recognized roles in testis/sperm function (File S1D). Finally, just under 2% of DML (*n* = 76) were common to both F1 and F2 sperm. Most were intergenic, but a cluster of four DML was located in intron 3 of *GGH* and 11 DML in intron 2 of *THRB*. There were also 12 genes harboring DML that were common to F1 and F2 sperm, one of which (*PTPRN2*) has a recognized role in testis/sperm function. In contrast to F1 sperm and seminal plasma, there were no DE miRNAs that reached the set criteria for FDR.

### F3 epigenetic effects—following EC exposure of pregnant F0 ewes

#### Liver DNA methylation

DNA was analyzed from 16 male and 16 female F3 offspring livers, balancing great-grandsire lineage across sex and treatment groups from the pool of available animals (Table S6). RRBS analyses covered 10,457,661 methylated cytosines. Hierarchical clustering of methylated CpGs again displayed strong separation by sex, with effects of treatment and great-grandsire lineage nested within sex (Fig. S3E). When partitioned by great-grandsire lineage, 20,116 DML were identified, 89.5% of which were specific to a single great-grandsire lineage (Fig. 3A) with 187 DML (138 genic) common to those in F2 sperm. When partitioned by sex, of 1,982 identified DML, 45.6 and 48.5% were specific to males and females, respectively (Fig. 3B). Additionally, 67 of these DML (58 genic) were common to DML in F2 sperm. In males, there was a net methylation increase of 2.0% (Fig. 3C), whereas in females, there was a net methylation decrease of 7.1% following BS exposure (Fig. 3D). In addition, 362 unique genes were identified as containing genic DML, with 51.7% specific to males and 44.8% to females (File S1I and J). Gene ontology (GO) analyses of these 362 genes identified 11 enriched “biological processes” terms in males (*P* ≤ 0.01) that encompass themes such as cell structure and adhesion, cell survival mechanisms, and development and differentiation (Fig. S5A). Females had 35 enriched GO terms with common themes of cell adhesion and migration, development, and morphogenesis and signaling (Fig. S5B).

**Figure 3:**
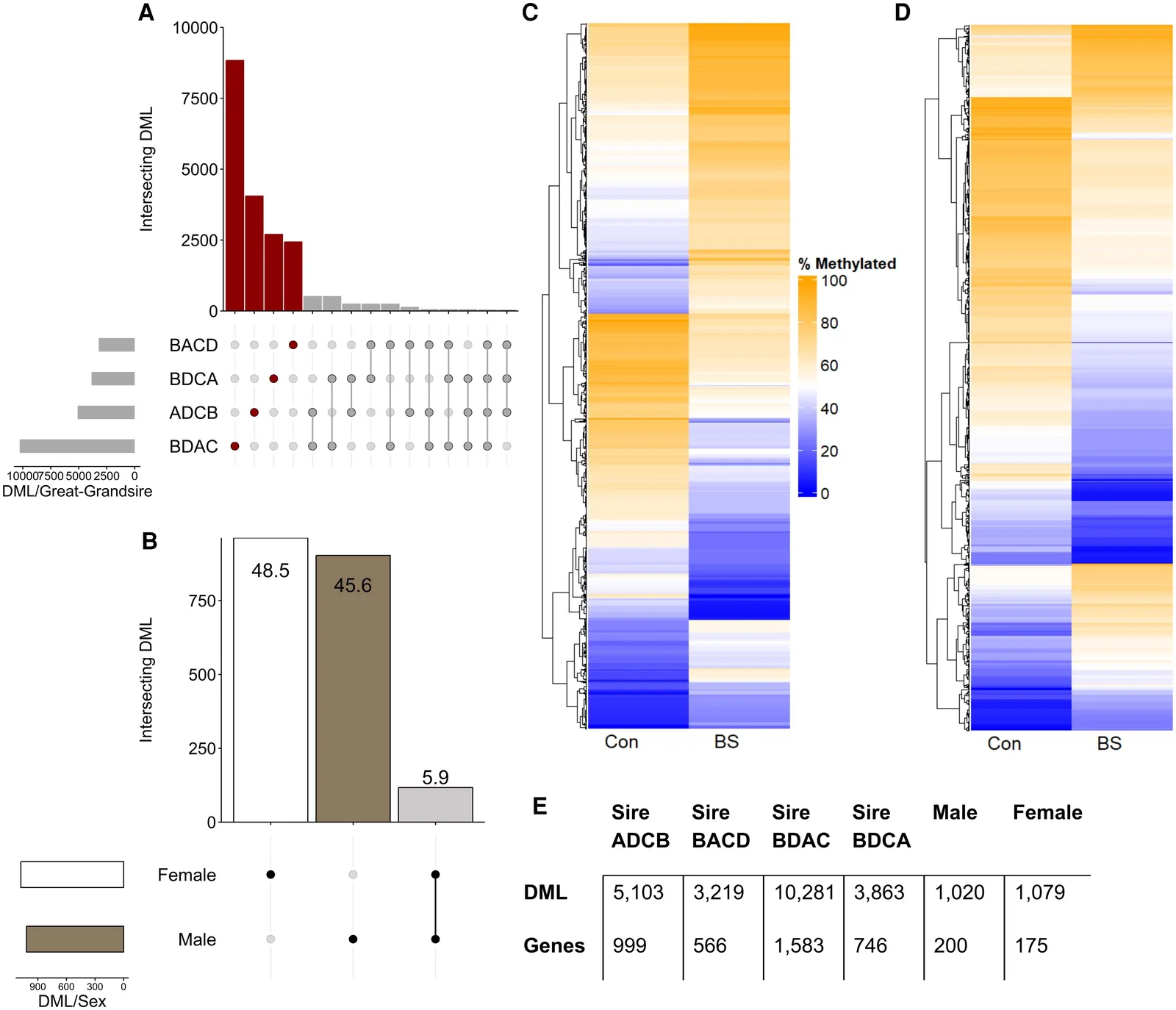
Methylation characteristics of F3 liver. A) Upset plot of DML (15% threshold) intersects across four F3 great-grandsire groups. B) Upset plot of DML intersects across male and female F3 liver (overlapping; values on bars denote percentage of total DML). C) Heat map highlighting mean methylation of DML in males in the control (Con) and BS exposed experimental groups. D) Heat map highlighting mean methylation of DML (in females in the Con and BS exposed experimental groups. E) DML and DML-containing gene counts across four great-grandsire groups as well as in males and females.

### Differential methylation across generations

These analyses focused on DML within F1, F2, and F3 livers. Initially, DML from each sex were compared across generations but no DML were found to be consistent across the three generations in either males (Fig. 4A) or females (Fig. 4B), other than a single genic DML region in a common and novel gene across the three generations in male (Fig. 4C) but not female (Fig. 4D) descendants.

**Figure 4:**
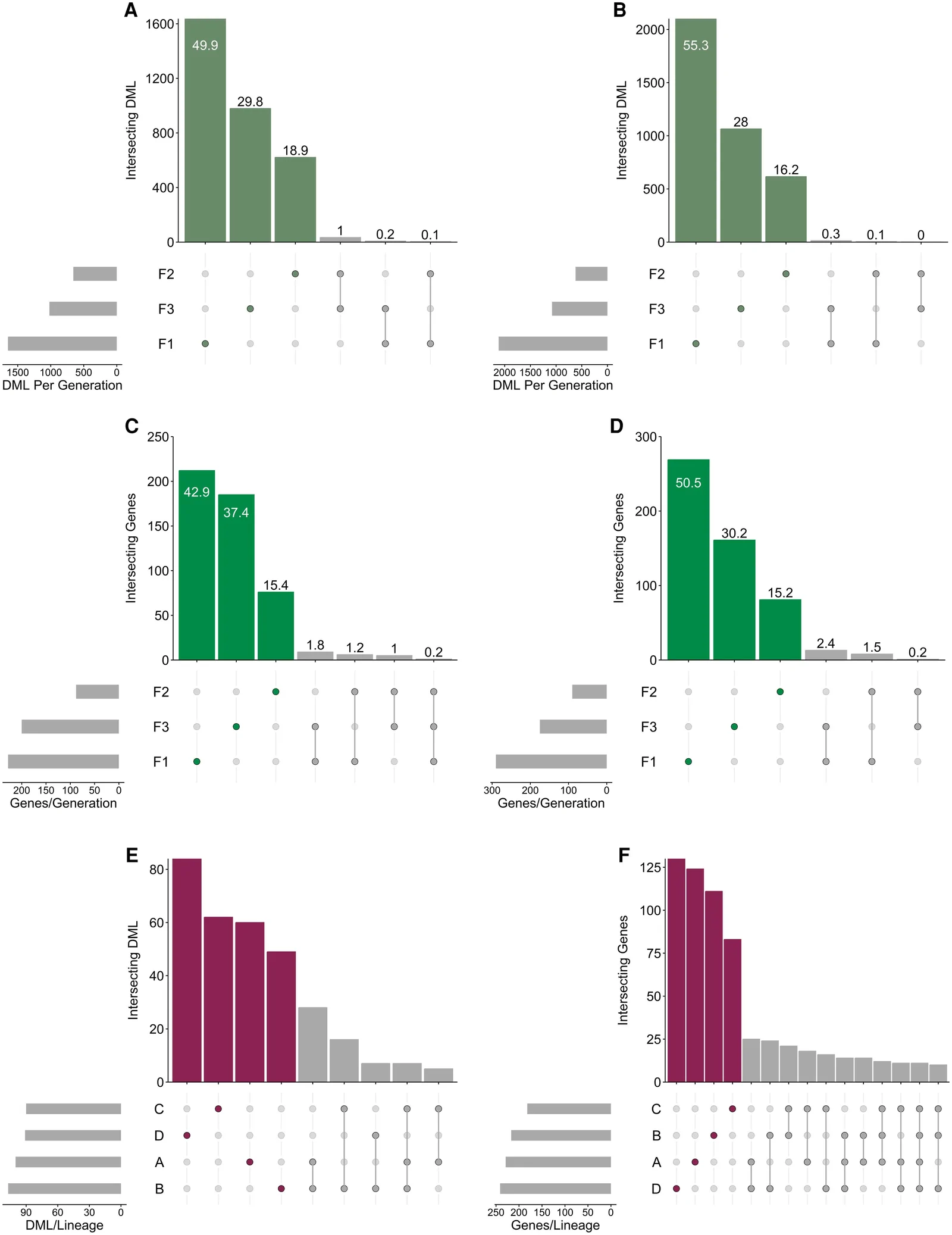
Intersecting DML and genes containing DML in liver samples across generations. A) Intersecting DML across three generations of male offspring. Values on bars denote percentage of total DML. B) Intersecting DML across three generations of female offspring. Values on bars denote percentage of total DML. C) Intersecting DML-containing genes across three generations of male offspring. Values on bars denote percentage of total DML. D) Intersecting DML-containing genes across three generations of female offspring. Values on bars denote percentage of total DML. E) Intersecting cross-generational DML from each lineage (e.g. tracking all instances of F0 Sire A from F1, F2, and F3 analyses partitioned by sire/grandsire/great-grandsire and averaged across sex), across the four lineages. F) Intersecting DML-containing genes present in all generations of a single lineage (e.g. tracking all instances of original sire A from F1, F2, and F3 analyses partitioned by sire/grandsire/great-grandsire and averaged across sex), across the four lineages.

An alternative approach partitioned analyses by sire (F1), grandsire (F2), and great-grandsire (F3) lineage, so that effects of sex within generation were averaged. This approach recognizes the significant proportion of DML that reside within descendants of each sire within each of the three generations (Figs. 1–3) and sought to track cross-generational DML within each founder-male lineage (i.e. descendants from F0 Rams A, B, C, and D) to identify DML present in all three generations of each lineage. Grandsire and great-grandsire analyses were collated according to original F0 ram sire to produce single F2 and F3 DML lists (e.g. for lineage A: F1 = A; F2 = AD, BA, DA; F3 = ADCB, BACD, BDAC, BDCA; Fig. S1). In total, 100 DML across 16 genes were found in lineage A, 107 DML across 17 genes in lineage B, 90 DML across 15 genes in lineage C, and 91 DML across 18 genes in lineage D. DML present in all three generations of a single lineage were then compared to those from other lineages to identify cross-generational DML that persisted regardless of lineage. None, however, were observed, although a seven-DML cluster within 39 bases within intron 3 of *DHRSX* (just downstream of 3′UTR of *ZBED1*; Fig. S6A) was consistent for all three generations across lineages A, B, and C (Fig. 4E). Adopting the same approach regarding genes containing DML, 11 genes were found to contain DML within all generations of all lineages. However, these DML were not consistent across generations within or across lineage. Instead, these genes (consisting of six known genes: *U6*, *MTCL1*, *PAQR5*, *ERG*, *SARDH*, and *MGLL*) represent common regions of the genome affected by differential methylation following BS exposure (Fig. 4F). Mapping DML for these six known genes revealed that, while they were generally clustered within each gene (e.g. DML within *PAQR5* were clustered within intron 1), the exact position did not match on all occasions.

Finally, a third analysis separated male and female descendants. For each sex, all samples from all three generations were analyzed. This involved averaging effects of sire, grandsire, and great-grandsire lineage (as presented in Fig. 4E and F), as well as effects arising from each generation. This identified 97 cross-generational DML in males (Fig. S7A) and 19 in females (Fig. S7B). DML positions in males and females were then compared between sexes (Fig. S7C), showcasing absolute sexual dimorphism in cross-generational DML. These analyses identified nine known genes (including *DHRSX*) and four novel (i.e. unannotated) genes in males. DML clusters were observed in four of these genes (i.e. *PGRMC1*, *5SrRNA*, *ME3/SNORD30*, and *DHRSX*). For *DHRSX*, eight DMLs clustered in intron 3, seven of which matched those identified cross-generationally in lineages A, B, and C (Fig. 4E). These analyses also identified two known (*DPP6* and *COL9A1*) and three novel genes in females. As *DHRSX* was identified in two separate analyses, transcript expression for this gene was determined in F3 liver. *DHRSX* expression was increased (*P* < 0.01) in F3 male (Fig. S7D) but not F3 female (Fig. S7E) descendants following in utero exposure of the F1 generation to chemicals originating from BS. This was consistent with a 28% decrease in methylation for all seven DML in intron 3 in F3 males with no change in methylation at the corresponding locus in females.

### “Escapees”

High-confidence demethylation-resistant genes within the germline (termed “escapees”) have been identified in the pig (31), a proportion of which have conserved synteny with both humans and mice (32, 33). From the published list of 101 escapees, 93 named candidates were cross-referenced to the list of hepatic F3 DML.

Initial analysis partitioned sex and averaged between great-grandsire lineages. Relative to regular genes, the odds ratio of escapees containing a DML was >5.1 (*P* < 0.01). However, only a few F3 DML within escapees were identified per sex, with none consistently represented across all three generations. Next, an analysis using DML obtained by averaging across sex and partitioning by great-grandsire lineage identified 219 DML in 30 escapees (Fig. S8A). In this case, the odds ratio of escapees containing a DML was significant (*P* < 0.001) but varied between great-grandsire lineages (ranging from 2.9 to 4.3). Then, averaging across sex, but including F1 and F2 generations along with F3, revealed 20 escapees (including *DPP6*) that contained DML identifiable across all three generations (Fig. S8C; File S1K). However, these DML were not necessarily the same in all three generations, although, importantly, further analysis did identify 10 DML that persisted across all three generations (Fig. S8B). Intriguingly, these DML all clustered within 169 bases of intron 4 of the tumor suppressor gene *CADM1* (Fig. S6B) with one DML in a region showing conservation with a human *CADM1* CCCTC-binding factor binding site involved in transcriptional regulation (34). Looking back at the F3 analysis partitioned by sex and averaged across great grandsires, these 10 DML were not detectable on average in either sex at the 15% threshold but present across F3 males only with a mean 9% increase in methylation. Importantly, transcript expression for *CADM1* was reduced (*P* = 0.043) in F3 male (Fig. S8D) but not F3 female (Fig. S8E) descendants.

Finally, germline cross-generational analyses of overlapping DML between F1 sperm and F2 liver and blood, and F2 sperm and F3 liver, for the listed escapees revealed 32 DML in five escapees in F1 sperm (Fig. S9A). In this instance, the two DML observed in sperm *DPP6* were also identified in both F2 liver and blood. These analyses also revealed 80 DML in five escapees in F2 sperm (Fig. S9B). Here, nine DML in sperm *DPP6* and six DML in sperm *COL5A1* were also identified in F3 liver.

## Discussion

Several important and novel findings emerge from the current study. First, we demonstrate that gestational exposure to a real-life mixture of EC, using an established outbred animal model of the human exposome (1), leads to measurable alterations to DNA methylation in F1 offspring, with detectable effects in F2 and F3 descendants. Equally, our data reveal the sizable extent to which genetic ancestry and offspring sex influence DNA methylation. Depending on tissue type, between 70 and 94% of DML within each generation were specific to a single sire lineage, and 88–96% of DML were sex specific. Such strong lineage and sex effects complicate interpretation and highlight the difficulty of distinguishing EC exposure-related epigenetic change from genetically regulated methylation variation (24, 25). The progressive convergence of F0 paternal lineage across generations (Fig. S1) was reflected in cytosine methylation clustering (Fig. 2), with sex and treatment effects becoming more pronounced in later generations. A key challenge is determining whether observed effects on DNA methylation constitute transgenerational epigenetic inheritance (TEI). In part, this requires (i) propagation of EC-induced epigenetic marks through the germline beyond the directly exposed generations, (ii) recurrence of the same EC-induced epimutations in the first unexposed generation (F3 in this study), and (iii) associated differences in gene expression (35). Based on these criteria alone, our data do not provide definitive evidence for TEI, although they highlight regions where environmentally associated methylation differences appear repeatedly across generations. Further analyses would be required to link observed DNA methylation differences in F1 and F2 sperm, and miRNA in F1 sperm and seminal plasma, with altered methylation in subsequent somatic-cell lineages. Furthermore, although persistently altered methylation was identified at specific loci, such as regions within *DHRSX* and *CADM1* (both of which were differentially expressed in F3 descendants), these changes were not uniformly present across all lineages. Such inconsistency, combined with RRBS's inherent genomic bias and the strong influence of allele-specific methylation (ASM) and methylation quantitative trait loci (meQTL) effects (36, 37), limits our ability to attribute these recurrent loci to stable transgenerational transmission rather than genetically driven variation. Nevertheless, these findings illustrate that complex, low-dose EC mixtures can induce robust methylation changes that recur in multiple generations in an outbred mammalian species and point to specific genomic regions where exposure-related perturbations arise repeatedly across generations, which warrant targeted investigation in future TEI-focused studies.

### DNA methylation sequencing in an outbred species

A key consideration in interpreting our findings is the sequencing approach used. RRBS enriches CpG-dense regions, which include CpG islands, promoter-proximal areas, and 5′ regulatory elements, via *MspI* digestion at 5′-C^CGG-3′ sites, which are overrepresented in these genomic contexts (38). In our study, this was reflected by the consistent distribution of hepatic DML between islands, shores, shelves, and open sea (43.0, 11.0, 5.2, and 40.5%, respectively). RRBS therefore provides a nonrandom and promotor-biased view, with limited coverage of distal enhancers and repetitive elements, regions that may be sensitive to environmental exposures (39). Instead, RRBS preferentially captures stable, cis-regulated CpGs and promoter-related methylation changes, which often reflect durable early-life programming effects (40) and have been implicated previously in developmental responses to toxicants in fish and mice (41–44). For the scale and given constraints of the current study, RRBS facilitated greater sequencing depth and biological replication across multiple generations and different cell types than would have otherwise been possible using alternative genome-wide single-nucleotide resolution platforms (45). Adequate depth and replication are essential in outbred species, where a substantial proportion of methylation variation is genetically influenced, including through ASM and meQTL (25, 36, 37). Our controlled breeding structure, which matched F0 sire lineages within and across generations, helped mitigate, but cannot eliminate, these confounding effects (46, 47). Filtering CpG overlapping known single-nucleotide polymorphisms (SNPs) further reduced artefactual differential methylation arising from CpG–SNPs (48). However, the effectiveness of this filtering depends on the completeness of available SNP catalogs and local linkage disequilibrium. Un-assayed causal variants in proximity to measured CpGs could still generate meQTL-like differences even when the CpG itself is invariant.

### Sexual dimorphism in DNA methylation inheritance

Several studies have reported sexually dimorphic DNA methylation responses following in utero and perinatal exposure to EC and heavy metals using RRBS in rodent offspring liver (43, 49, 50) and heart (51). In our study, the magnitude of sex-specific differences in hepatic DNA methylation across generations following in utero EC exposure was comparable to the influence of sire lineage itself. Notably, there is no evidence that RRBS inherently exaggerates sex differences compared with other single-nucleotide resolution platforms (52). As RRBS captures CpGs mainly within promoter-proximal and CpG-island regions, while incompletely sampling distal enhancers, intronic elements, and open-sea regulatory sites (53), our findings should be interpreted primarily within this regulatory context. Many hepatic genes with stable, sex-biased methylation patterns in mammals, including those regulating lipid metabolism, energy homeostasis, and endocrine pathways (e.g. *IGF1*, *NR3C1*, and *PPARA*), are influenced by methylation in these promoter-proximal regions (54–56). Although these canonical loci did not feature in our cross-generational gene sets, 68 other metabolic genes did (File S1). Most were male-biased, with only nine expressed in females. Among these, *IMMP1L*, *TFPI*, and *DPP6* contribute to hepatic proteolysis and nitrogen metabolism, while *GALNT17*, *DEPTOR*, *PDZRN3*, and *EEFSEC* regulate transcription, translation, or protein synthesis. Functional enrichment further highlighted divergence between sexes: male-biased genes were enriched for processes such as cell adhesion, apoptosis, oxidative phosphorylation, and carbohydrate utilization; whereas female-biased genes were more strongly associated with developmental signaling pathways (e.g. NOTCH, wingless-related integration site [WNT], and bone morphogenetic protein [BMP]), lipid differentiation, and peroxisomal dynamics. Both sexes shared enrichment for homophilic cell adhesion, suggesting a conserved biological theme achieved through sex-specific molecular networks. These sexually dimorphic effects could have arisen because of sex-chromosome-directed epigenetic alterations to autosomal DNA methylation and/or due to the actions of sex steroids (26). Given that EC exposure extended throughout gestation, it is not possible at this juncture to ascertain which of these mechanisms may have been more prominent.

### Intergenerational epigenetic inheritance

In utero exposure to endocrine disruptors and other toxicants has altered the profile of small noncoding RNA of adult F1 sperm in laboratory species (57–59). Furthermore, semen-borne miRNA can contribute to paternal epigenetic signaling, consistent with recognized modes of intergenerational inheritance, although mechanistic details remain uncertain (60). Notably, while gestational toxicant exposure can induce sperm miRNA differences that persist to F3 in outbred rats (61), the six annotated sheep miRNAs detected here were restricted to F1 semen. One of these, miR-539-5p, is known to regulate DNA methylation in mice by targeting *Dnmt3b*, thereby modifying promoter CpG-island methylation and transcription of lipogenic genes such as *Srebf1* (62). While no comparable role has been defined for the remaining five microRNA, miR-544-5p forms part of a cluster of miRNA within the DLK1-GTL2/MEG3 imprinted domain on sheep chromosome 18, with miR-376b-5p, miR-376e-5p, and miR-376d residing close by (63). This domain regulates the callipyge muscle hypertrophy phenotype in sheep (64, 65). However, no differential methylation at this locus was detected in our somatic-cell datasets (File S1), consistent with its muscle-specific regulation (65).

The current study identified a number of overlapping DML between F1 sperm and F2 liver or blood and between F2 sperm and F3 liver, an observation consistent with the fact that some genomic regions escape epigenetic reprogramming and can retain methylation information across germline and somatic lineages (32, 66). However, care is required when ascribing these as inherited differences. The current dataset cannot distinguish true inheritance from coincidental coverage or meQTL-driven methylation. Of the genes listed as harboring overlapping DML (File S1), however, *SNED1* (with 45 overlapping DML) and *DPP6* (a listed escapee with nine overlapping DML) are worthy of further investigation. Also, *PTPRN2* (Fig. S8A) is another listed escapee that overlapped between F1 and F2 sperm. Although it did not feature in the list of DE genes for the testis in the current cohort of F1 animals (12), hypermethylation and reduced expression of this gene in sperm are associated with reduced sperm counts and motility in men exposed to high levels of cigarette chemicals (67, 68).

When broader functional sets were examined, pairwise comparisons of differentially methylated hepatic genes (File S1) across generations revealed consistent enrichment for “cell organization,” “cell adhesion,” and “lipid/redox metabolism” in males. Intriguingly, plasma and liver triglyceride concentrations were both increased in males, but not females, among the broader cohort of our participating F1 offspring (21). In females, overlapping gene sets were enriched for cell adhesion, “neurodevelopmental signaling,” and “transcriptional regulation.” Although underlying DML and gene identities varied between generations, the stability of these functional themes suggests that intergenerational liver responses may be conserved at the process level rather than through persistence of identical epimutations.

A key criterion for TEI relates to the inheritance of the same epimutation(s) across generations extending to the first unexposed generation (F3 in this study) (35); although this requirement could be considered too restrictive, reflecting a DNA methylation-centric view of inheritance. Indeed, emerging evidence indicates that germline epigenetic information is encoded within an interacting network of DNA methylation, retained histones, and noncoding RNAs (69), thus preserving regulatory information across generations while allowing the precise genomic location of altered cytosine methylation to differ between generations. Nevertheless, identical DML persistence across generations provides strong evidence of direct epigenetic mark inheritance. Two genes from our dataset warrant consideration in this respect. The escapee *CADM1* harbored 10 DML clustered in a conserved intronic region of the gene, which, in humans, is associated with transcriptional regulation (34). This cluster was detected in at least one lineage across all three generations (Fig. S6B), and increased methylation in F3 males was associated with reduced transcription (Fig. S8D). *CADM1* is a recognized tumor suppressor gene regulating hepatic cell-cycle progression (70) and mediating endothelial adhesion and liver inflammation (71). Interestingly, prenatal testosterone exposure in adult female sheep altered methylation at a separate intronic DMR of *CADM1* (located in intron 2) but with no effect on transcription (72), supporting its sensitivity to developmental endocrine perturbation. The second gene, *DHRSX*, is involved in the dolichol pathway, linked to glycosylation and hepatic glycoprotein production (73). Importantly, it is located in the pseudo-autosomal region of the X-chromosome in both humans and sheep (74, 75) and thus escapes inactivation. We identified seven DML clustered within intron 3 (Fig. S6A) that recurred in all three generations in lineages A, B, and C (Fig. 4E), although the underlying cause cannot be distinguished. Furthermore, reduced methylation at this locus in F3 males aligned with increased transcript expression (Fig. S7D). This methylation pattern was consistent across all generations of males in lineages B and C but not in lineage A, suggesting partial genetically inherited regulation of methylation at this site. A putative imprinting control region located in human *DHRSX* intron 3 (76), which appears orthologous to the sheep locus, raises the possibility of parent-of-origin effects contributing to the observed sexual dimorphism.

### Concluding remarks

This study demonstrates that gestational exposure to low-level complex mixtures of EC, representative of the human exposome, can induce sexually dimorphic and lineage-specific changes in DNA methylation that can recur across multiple generations in an outbred large-animal model. Although no single DML persisted consistently in all lineages across all generations, the identification of recurrent changes in regions within defined genes such as *DHRSX* and *CADM1* provides intriguing, although not definitive, evidence for multigenerational recurrence of exposure-induced epimutations. These findings underscore the challenge of disentangling true transgenerational DNA methylation inheritance from underlying genetic lineage-directed variation. Missing from the current dataset are metabolic and reproductive phenotype data linked to DML for F2 and F3 descendants. These are required to demonstrate transgenerational inheritance (35) and are the subject of ongoing investigations with the current cohort of animals. Conceptually, however, findings from our study concur with the conclusion reached, following a series of TEI studies in sheep, that genomic context is a key determinant of how methylation changes persist across generations following initial environmental exposure (77–79). Consequently, future research will expand our initial observations to whole-genome approaches to capture distal enhancer and repeat sequences. Germline transmission analyses will be expanded to include oocytes and to assess chromatin accessibility using contemporary single-cell multiomic approaches. Dense genotyping combined with whole-genome sequencing will map meQTL and ASM, testing methylation using pedigree-aware mixed models to partition genetic and exposure effects. Ultimately, however, further reciprocal matings across lineages may be required to disentangle sire/grandsire contributions and assess DML persistence within haplotypes.

## Materials and methods

Full details can be found in SI Appendix, Detailed Materials and Methods. Animal procedures were approved by the Animal Welfare and Ethical Review Board of the University of Glasgow and performed under the United Kingdom's Animals (Scientific Procedures) Act 1986 (licensed authority: PF10145DF). Protocols complied with the ARRIVE guidelines.

### Experimental treatments, animals, and design

EasyCare ewes (*Ovis aries*; F0, *n* = 320) were allocated randomly to graze either Con or BS treated pastures at the University of Glasgow Cochno Farm and Research Centre (7) from 6 weeks prior to mating (by AI) until lambing, then grazed on Con pastures thereafter. AI (80) was undertaken in 307 F0 ewes using semen from four unrelated F0 rams (labeled A, B, C, and D) not exposed to BS, generating four F1 family groups matched across treatments (Fig. S1, Table S1) (15). Subsequently, semen from eight F1 rams (four Con and four BS), from each of the four families within treatment group, was used for AI of 149 F1 ewes from the other three families (Fig. S1, Table S2). Natural matings between F2 rams and ewes (*n* = 61) were undertaken within each treatment group to generate F3 offspring (Fig. S1, Table S3).

### CASA and semen cryopreservation

Semen was collected from 16 F1 and 14 F2 sexually mature rams on two occasions within generation, balancing sire lineage between treatments (Table S1). Sperm analyses were undertaken using iSperm mCASA (Aidmics Biotechnology Co., Ltd, Taipei, Taiwan) (81). Residual seminal plasma and sperm were stored at −80 °C.

### miRNA analyses from sperm and seminal plasma

Following extraction (miRNeasy kit; Qiagen) with DNase treatment (82) sperm RNA libraries were pooled and sequenced on the Element Biosciences AVITI System on an AVITI 2X75 Sequencer—Cloudbreak FS Medium Output (Element Biosciences; 860-00014), generating ∼20 million 75-bp single-end reads per sample. Raw sequencing reads were aligned to mature and precursor sheep miRNA sequences from the miRBase database (83). DE performed on miRNAs with >10 counts per million using the Bioconductor edgeR package v4.0.16 (84). DE miRNAs = log fold change ≥2.0, FDR ≤0.01 (85).

### DNA extraction

DNA was extracted from liver/blood using DNeasy Blood and Tissue Kit (Qiagen) with RNase A and from sperm using gradient plus somatic-cell lysis (with DNase) followed by salting-out (86).

### Reduced-representation bisulfite sequencing

DNA and unmethylated λ (Promega, Southampton, United Kingdom) were digested with *Msp1* (NEB, Hitchin, United Kingdom). The methylated adaptor was ligated to DNA. RRBS libraries were then prepared using bisulfite-converted DNA. Size-selected fragments (200–600 bp) were pooled and sequenced (Illumina NovoSeq X plus), >80 million 150 bp pair-end reads per sample (Novogene, Cambridge, United Kingdom).

### Differential methylation analyses

Sequenced RRBS libraries were aligned to the Ensembl reference genome ARS-UI_Ramb_v2.0 (GCA_016772045.1). CpGs matching positions of known SNPs were filtered. Analyses were performed using DSS (87) with a threshold ≥15% difference in methylation, FDR ≤0.01 (85). Identified DMLs labeled as genic or intergenic using the ChIPseeker v1.34.1 package (88, 89). GO and Kyoto Encyclopedia of Genes and Genomes (KEGG) pathway analyses were performed on genic DMLs. Escapees (31) were identified and enrichment determined using Fisher's exact test.

### Statistical analysis

Used GenStat statistical package (21st Edition, VSN International, 2022; https://www.vsni.co.uk/). Sperm motility and transcript expression (F3 livers) were analyzed using REML generalized linear mixed models (fixed term = treatment; random terms = lineage group [F1, F2, or F3]). Data are presented as predicted means ± SEM.

## Supplementary Material

pgag338_Supplementary_Data

## Data Availability

All study data are included in the article, SI Appendix, and Data File S1. RRBS and RNA sequencing data have been deposited in the European Nucleotide Archive and are accessible through accession number PRJEB109928 https://www.ebi.ac.uk/ena/browser/view/PRJEB109928.

## Preprints

This manuscript was posted on a preprint at 10.64898/2026.04.08.717152.
